# Motion Driven by Strain Gradient Fields

**DOI:** 10.1038/srep13675

**Published:** 2015-09-01

**Authors:** Chao Wang, Shaohua Chen

**Affiliations:** 1 LNM, Institute of Mechanics, Chinese Academy of Sciences, Beijing 100190, China

## Abstract

A new driving mechanism for direction-controlled motion of nano-scale objects is proposed, based on a model of stretching a graphene strip linked to a rigid base with linear springs of identical stiffness. We find that the potential energy difference induced by the strain gradient field in the graphene strip substrate can generate sufficient force to overcome the static and kinetic friction forces between the nano-flake and the strip substrate, resulting in the nanoscale flake motion in the direction of gradient reduction. The dynamics of the nano-flake can be manipulated by tuning the stiffness of linear springs, stretching velocity and the flake size. This fundamental law of directional motion induced by strain gradient could be very useful for promising designs of nanoscale manipulation, transportation and smart surfaces.

Transporting an object from one place to another is a basic and ubiquitous activity in nature. From the ancient Egyptian carrying large stone to build pyramids to modern rockets conveying several hundred tons of goods far away from Earth, transporting objects much heavier than human weights is always a huge challenge, special tools and methods must be resorted to. In the mysterious nano-world, exploring nanoscale mechanisms in nano-bio-systems^1^,^2^ or new materials^3^ is another challenge for the development of advanced science. How to manipulate or transport nanoscale objects accurately becomes an inevitable task nowadays, which is vitally important in both engineering and science of NEMS^4^ and bio-systems^1^. How to drive a nanoscale object move directionally on a surface or in a tunnel has attracted increasing interests in recent years. Many methods have been proposed to induce nanoscale motion by different mechanisms, including techniques of applied voltage^4^,^5^,^6^, electric current^7^,^8^, thermal energy^9^,^10^,^11^,^12^,^13^, biological processes^1^, asymmetrically chemical interactions^14^,^15^,^16^,^17^, stiffness gradient substrate^18^ or noise-assisted Brownian motors^19^,^20^,^21^,^22^. However, how to realize nanoscale manipulation and transportation in different environments with different requirements still needs more intensive researches.

Cell motion tuned by substrate strain is a well-known transportation in micro-scales^23^. Raeber *et al.*^23^ found experimentally that fibroblasts migrate preferentially in the direction of principal strain. An interesting finding in recently experimental and theoretical studies^24^,^25^ show that strain gradient induced by the smooth muscle contraction of fallopian tube may be one of the primary mechanisms to drive oosperms rolling along the fallopian tube. Comparatively, in the early study of crystal dislocations^26^, it was found that the inhomogeneous strain field around dislocations in a crystal would cause solute atoms to diffuse from one region to another. However, to our best knowledge, no explicit report and feasible nano-technology on strain gradient as a driving force to realize nano-motion exist.

Non-equilibrium molecular dynamic simulation is carried out in the present paper in order to disclose whether a nanoscale object could be driven by the strain gradient in substrates. An inspiring result is achieved that a nanoscale flake on a graphene strip substrate would move spontaneously from the region with higher strain to the one with lower strain.

The simulation setup is shown in Fig. 1, where a stretchable single-layer graphene strip with armchair edges along the *y* axis is attached on a rigid base. A short graphene slider (nano-flake) with armchair edges along the *y* axis is placed on the top of the graphene strip as illustrated in Fig. 1(a). A commensurate geometry between the slider and the graphene strip substrate is adopted with open boundary conditions in the *y* direction. Both the stretchable graphene strip substrate and the rigid base are 50 nanometers long and 2.5 nanometers wide. The nano-flake is 1 nanometer long and 2.5 nanometers wide. The distance between the nano-flake and the graphene strip is 0.34 nm, an equilibrium separation. Relative to the small graphene slider, the underlying graphene strip is called as strip substrate in the present model. In order to introduce strain gradient in the graphene strip substrate, carbon atoms in the strip substrate are linked to the rigid base by linear springs with identical stiffness as shown in Fig. 1(b). The strip substrate is stretched in the x-direction by imposing a constant velocity to atoms at the right end of the strip. During stretching, the spring will break as its length exceeding a critical value, inducing an interface crack between the substrate and the rigid base. A strain gradient field would appear in the substrate ahead of the crack tip. The simulation shows that the small slider moves spontaneously in the direction of strip substrate strain reduction when the crack tip reaches the location of the slider [Fig. 1(c)] (details of the methodology can be found in the Supplemental Material). In the present technology, the interface between the strip substrate and the rigid base is a solid one with a strength, which is simulated by linear springs. An interface crack will emerge when the external force is large enough to break interface springs. It is not healable, unlike a biological defect. In all our simulations, the critical length of interface springs is 0.4 nm, independent on the value of their stiffness. The maximum strength of each spring is *Kδ*^2^/2, where *K* is the stiffness of the spring and *δ* is the critical length, similar to the Tvergaard-Hutchinson model^27^.

Without interface springs, we tension uniformly the graphene strip substrate at its right end with its left-end fixed, uniform strain will be produced in the strip without gradient. In real applications, several methods can be used to introduce bond cross-links between the graphene strip substrate and the rigid base, including moderate electron-beam irradiation^28^ or adding chemical group^29^.

In order to observe a periodic movement of the slider on the strip substrate with a finite distance, a viscous force proportional to the slider velocity is exerted on the slider atoms to consume its kinetic energy. The movement of the slider and the stretchable substrate are restricted only in the *x*-direction, any other effects are eliminated, such as the slider twist, the substrate fluctuation and the Poisson’s effect.

Figure 2(a) shows some typical snapshots of the slider moving along the substrate (see Movie 1 in the Supplemental Material). The slider is initially placed at the right end of the strip substrate. During the relaxation process without external extensions, the slider accelerates due to the non-equilibrium force^30^,^31^ exerted by van der Waals force, and then decelerates due to the effect of the viscous force. Finally, it reaches a position ahead of the right end of the strip substrate and oscillates near an equilibrium position with a decreasing velocity. As a constant velocity is added on the atoms at the right end of the graphene strip, both the lattice size of the graphene strip in the *x*-direction and the length of underlying springs linked to the rigid base will increase. When the length of springs exceeds a critical value, the spring will break. An interface crack initiates and then propagates towards the left. The left end of red-colored atoms as shown in Fig. 2(a) denotes the position of the crack tip. A graded strain field exists in the strip substrate ahead of the crack tip, which is demonstrated by gradient colored atoms. The motion of the slider is triggered as soon as the crack tip arrives at the slider, and it stops somewhere ahead of the crack tip. As the strip is pulled continuously at its right end, the crack tip will catch up with and activate the slider once more. Such a repeated motion clearly shows that the strain gradient in the strip substrate ahead of the crack tip offers a driving force on the slider.

Quantitatively examination on the strain gradient ahead of the crack tip is shown in Fig. 2(b). The maximum strain gradient in the substrate attains 0.45 nm^−1^ and appears near the crack tip, which decreases exponentially to zero at the distance of about 2.5 nm ahead of the crack tip. Behind the crack tip, the strain gradient is nearly zero, which indicates a uniform deformation in the pulled-off part of the graphene strip. Amplitude of the strain gradient in the substrate should depend on the stiffness of springs linked to the rigid base, i.e., the interface strength. The higher the stiffness of the spring, the larger the strain gradient in the substrate is, which can be found in Fig. 2(b).

Velocities of the slider and the crack propagation as well as the driving force exerted on the slider are extracted quantitatively [Fig. 3(a)]. The interface crack initiates at about 50 ps and propagates at a nearly constant velocity 0.05 nm/ps, which is nearly 5 times of the stretching velocity. The maximum velocity of the slider is about 0.15 nm/ps, which is 0.1 nm/ps larger than the constant one of the crack tip. The slider moves forward alone and stops somewhere due to the viscous force. Meanwhile, the crack tip propagates continuously due to the external loading and finally gets to the location of the slider. The resting slider moves again for the second time, and so on. The period of the slider motion is about 130 ps. The larger the external stretching velocity, the faster the crack propagates. When the velocity of the crack tip exceeds that of the slider, it will leave the slider behind and the slider will not be activated any more due to a uniform deformation in the pulled-off graphene strip (see Movie 2 in the Supplemental Material). The largest driving force originated from the strain gradient ahead of the crack tip is about 60 pN when the stiffness of springs is 100 eV/nm^2^. The stiffness of springs can be tuned precisely. A larger spring stiffness would induce break at the right end of the graphene strip itself and a smaller one could not generate an enough driving force to overcome the viscous resistance. Our simulation shows that the motion of the slider can be induced when the spring stiffness is in a range of 100–500 eV/nm^2^ (16–80 N/m). In real experiments, the interface strength may be influenced by interfacial impurities or defects, which, however, can be tuned with binders of different strength.

The periodic variation can also be identified from the conversion of the kinetic energy and the potential one of the slider as shown in Fig. 3(b). When the crack tip approaches the slider at some moment, the slider does not move immediately but keeps motionless for a short time, during which the potential energy increases. As the movement of the slider is triggered, the potential energy will decrease with a corresponding increase of the kinetic energy. The variation of the potential energy is about 5 meV/atom, which is larger than that of the kinetic energy 1.25 meV/atom, indicating the energy dissipation in the sliding process. In fact, the mechanical energy inputted from the outside is transferred to the strain energy stored in the pulled-off part of the graphene substrate, the strain energy stored in the strain gradient region ahead of the crack tip, the energy needed by new surfaces forming at the interface between the graphene substrate and the rigid base, as well as the potential energy of the graphene nano-flake. Only when the potential energy of the graphene flake achieves some value in the strain gradient region, it will be transferred to the kinetic energy. As a result, the graphene flake moves with the resistance of interface friction. It can be inferred that most of the mechanical energy inputted from the outside in such a system is transferred to the first three kinds of energies, only a very small part is converted to the potential energy of the nano-flake. Part of the potential energy is then transferred to the kinetic one of the nano-flake and part of the potential energy is dissipated due to the interface friction between the nano-flake and the graphene substrate. For example, the energetic efficiency in the first period of motion in the case shown in Fig. 3b, i.e., in the region of 70–200 ps, the total inputted energy is about 832 eV and only 0.01% is transferred to the kinetic energy of the nano-flake. However, the energetic efficiency may be large if we adopt another technique to produce strain gradient fields, for example, laterally non-uniform tension of a strip. The main aim of the present paper is to investigate the driving mechanism in nanoscale by strain gradient fields.

The driving mechanism of the motion guided by the strain gradient of the substrate can be further elucidated by the motion of the slider when the substrate is subjected to a uniform tension without the restriction of a rigid base. The slider is initially placed at the center of the substrate. No motion can be found for the slider due to a zero strain gradient in the substrate. As shown in Fig. 4, the corresponding potential energy of the slider increases almost linearly with the substrate strain, without conversion of the potential energy and the kinetic one.

According to the finding in Fig. 4, the larger the substrate strain, the higher the potential energy of the slider is. When a segment of the slider lies in a higher strain region, the part of the slider should possess a higher potential energy than other segments of the slider. It means that the strain gradient in the graphene substrate would induce a gradient of the potential energy, which drives the motion of the slider. On the contrary, if the slider is placed in a uniform strain field, the gradient of the potential energy vanishes and the slider will remain motionless. From the thermo-mechanics point of view, a lower potential energy means a more stable state that an object prefers to. That is the reason of why the slider moving towards a region with a smaller deformation.

Furthermore, the size of the slider itself should also be chosen carefully to ensure the motion. In cases with a given stiffness of springs, the cohesive zone (strain gradient field) ahead of the crack tip has a critical length, e.g. about 2.5 nm in the simulation shown in Fig. 2(b). A slider much shorter than the cohesive zone could hardly feel the strain gradient, resulting in a motionless slider. In addition, if the size of the slider is much larger than that of the cohesive zone, the driving force induced by the strain gradient will lead to the motion of the slider. But the velocity is very small due to a large interface resistance exerted to the large slider. The crack tip will exceed the slider when the slider moves a relatively short distance. After that, the slider will be left on the pulled-off graphene strip and stay there without activation (details of behaviors of a short slider and a large one can be found in Figs S1 and S2 in the Supplemental Material, respectively). It can be found that, with a determined external loading velocity, the larger the slider, the more slowly the slider moves.

The driving force induced by the strain gradient in substrates can be quite large in contrast to that resulted from the gradient of other fields. For instance, a strain gradient of 0.4 nm^−1^ in our simulation shown in Fig. 2(b) can generate a force of 60 pN on the nano-flake of a length 1 nm in the tension direction and a width 2.5 nm (about 24 MPa per unit area). For comparison, a thermal gradient of 1 K/nm can generate a force of about 5 pN on a nanotube of a diameter 2 nm and a length 8 nm (about 100 kPa per unit area)^32^. A stiffness difference between 0.801 and 2.403 can generate a force of about 4 pN on a 6 nm wide slider (about 320 kPa per unit area)^18^.

The strain gradient in substrates is realized by an interface crack in the present work, which can also be designed directly in some elementary nano-structural materials, for example, nanotubes^33^ or graphene strips^34^, by tuning their microstructures with gradient distributed dopants. Thus, a smart surface or structure with a gradient internal strain can induce spontaneous nano-driving, which can further serve as an on-line controllable nano-manipulation and transportation technology.

In summary, non-equilibrium molecular dynamics simulation is carried out to investigate motion of a nano-flake on a graphene strip subjected to a graded strain. A new feasible scheme using non-homogeneous strain to induce and manipulate the motion of nano-objects is proposed. The underlying physics is that the slider on the substrate with higher strain possesses higher potential energy and a gradient strain field would induce a graded field of the potential energy, which serves as a driving force for motion. The scheme is realized in the present work by stretching a graphene strip linked on a rigid base with numerous springs, inducing an interface crack, ahead of which a strain gradient field can be fabricated spontaneously. A smart surface or structure can also be designed to get an intrinsic capability for nano-object motion. The finding offers a novel idea for manipulating and transporting nanoscale particles in many fields, for instance, bio-nano-systems and NEMS, and a new approach to design smart and multifunctional surfaces for nanoscale directional motion.

## Additional Information

**How to cite this article**: Wang, C. and Chen, S. Motion Driven by Strain Gradient Fields. *Sci. Rep.* **5**, 13675; doi: 10.1038/srep13675 (2015).

## Supplementary Material

Supplementary Information

Supplementary Movie 1

Supplementary Movie 2

## Figures and Tables

**Figure 1:**
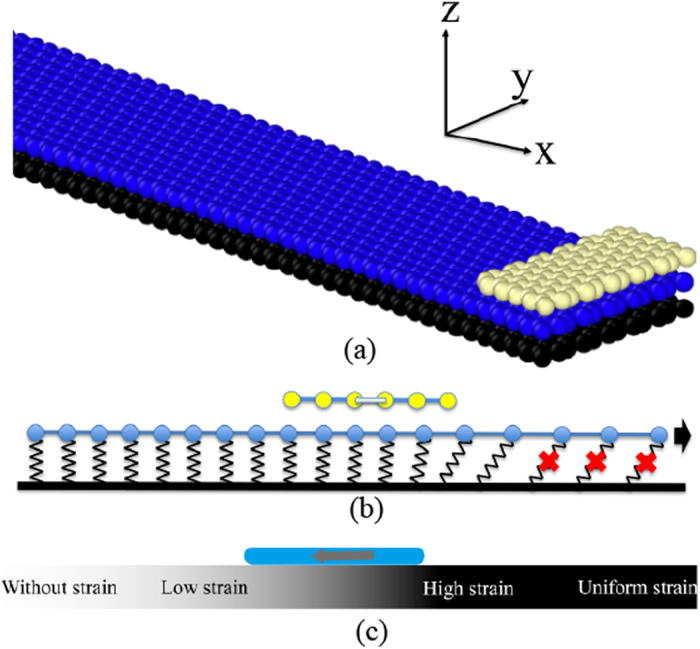
The molecular dynamics simulation model of the slider/graphene strip substrate/rigid base system. (**a**) Configuration of a small graphene slider lying on a long graphene strip substrate. (**b**) Schematic of the slider moving on the stretched strip substrate, which is linked to a rigid base through identical springs. (**c**) Schematic of nano-flake motion from a higher strain region to the one with a smaller strain.

**Figure 2:**
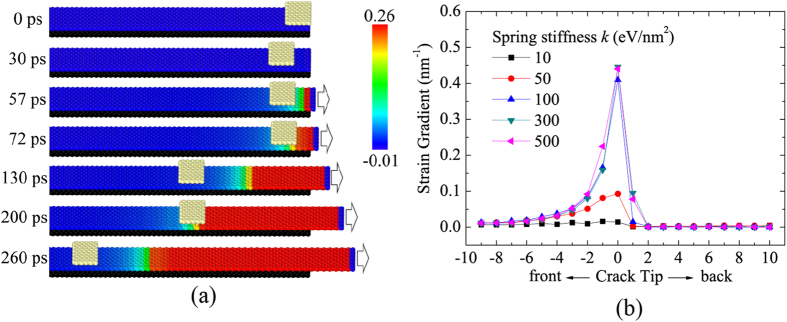
(**a**) Typical snapshots of the slider movement on the graphene substrate during stretching. The slider is initially put at the right end of the graphene strip at 0 ps; Due to the non-equilibrium force induced by the van der Waals one, the slider moves and finds an equilibrium position at 30 ps; After that, an externally tensile force is added on the right end of the graphene strip, which initiates an interface crack between the graphene strip and the rigid base at 57 ps; The crack tip arrives at the position that the slider stays at 72 ps; The slider moves forward quickly and finds another equilibrium position at 130 ps; The crack tip comes close to the slider once more at 200 ps; The slider moves again with a faster velocity than that of the crack tip at 260 ps. (**b**) Distribution of the strain gradient field in substrates for cases with different spring stiffness. Here, the spring stiffness can be regarded as the interface strength.

**Figure 3:**
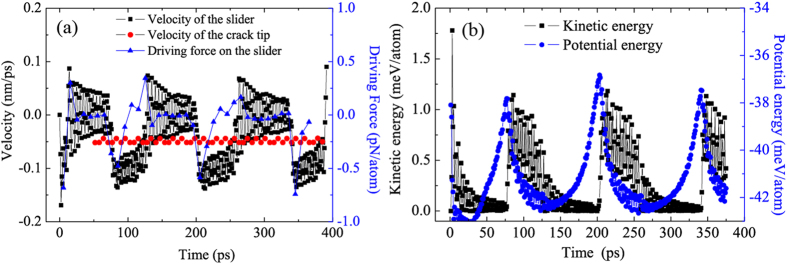
(**a**) Velocities of the slider and the crack tip as well as the driving force of the slider as a function of the simulation time. Initially, the slider is put on the right end of the graphene strip. Due to the non-equilibrium force yielded by van der Waals one, the slider starts to accelerate and achieves the maximum velocity, after which the velocity reduces gradually until zero. Then the slider moves in a contrary direction, whereafter oscillating near an equilibrium position. An interface crack initiates and propagates with a constant velocity due to an external force added on the right end of the graphene strip. When the crack tip comes close to the position below the slider, the slider starts to accelerate due to a driving force induced by the strain gradient in the graphene strip and achieves the maximum, then the velocity of the slider decreases gradually until zero. The slider moves in a contrary direction due to a relatively high potential energy, after which it will oscillate near another equilibrium position. At this time, the driving force is nearly zero due to the crack tip far away from the slider. When the crack tip comes close to the slider once more, all the phenomena happen cyclically. (**b**) Conversion between the kinetic energy and the potential one of the slider. When the slider lies at an equilibrium position, the potential energy of the slider increases continuously due to the more and more closer strain gradient field. When the potential energy achieves the maximum, the slider will move, which leads to a sharply decreasing potential energy and a suddenly increasing kinetic one.

**Figure 4:**
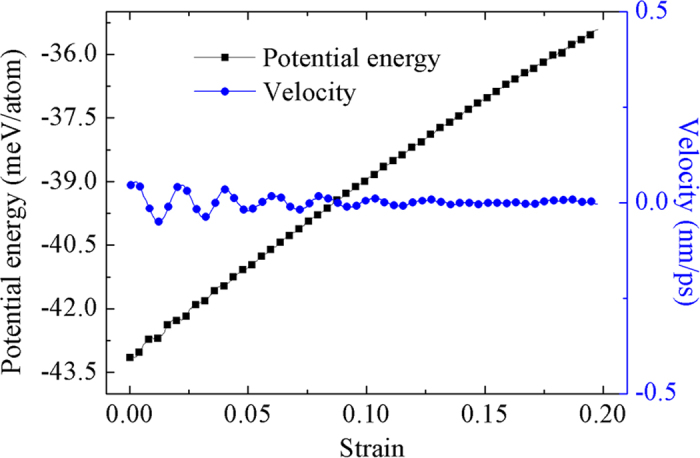
The potential energy and the velocity of the slider as a function of the uniform strain in the substrate. If an external force is added at both ends of a free graphene strip, the strain in the graphene strip is uniform and increases with the external force. The potential energy of the slider lying on the stretched strip increases almost linearly with the increasing strip strain. However, a uniform strain field in the graphene strip cannot induce motion of the slider only if the slider stays at an equilibrium position.
