# Supplementary material for: Motion Driven by Strain Gradient Fields
Source: Sci Rep. 2015 Sep 1;5:13675. doi: 10.1038/srep13675 (PMC4555178; doi:10.1038/srep13675)
Supplement: Supplementary Information [file srep13675-s1.doc]

Supplemental Material for the paper " Motion Driven by Strain Gradient Fields" written by Chao Wang and Shaohua Chen

**Methodology**

The length of the nano-flake changes from 0.5 nm to 20 nm in our simulations in order to study the size effect of movement. The stretch velocity added on the strip substrate changes from 0.001 to 0.1 nm per picosecond in order to investigate the effect of crack propagation velocity on the slider behavior. A viscous force with a viscosity 10 eV·ps/nm^2^ is added to atoms in the graphene strip to reduce oscillations during the stretch process. Another viscous force proportional to the velocity of the slider is added to atoms in the slider in order to simulate interfacial nano-frictions. In all our simulations, a reasonable value of viscosity 0.1 eV·ps/nm^2^ is taken.

**Movies**

Movie 1. The whole process of a slider of 1 nm long and 2.5 nm wide moving on a graphene strip substrate linked on a rigid base by springs. The stretch velocity added at the right end of the graphene strip is 0.01 nm/ps and the stiffness of springs is 100 eV/nm^2^.

Movie 2. The effect of a relatively high stretch velocity 0.1 nm/ps on the slider behavior. After relaxation, the slider stops at about 1 nm away from the right end of the substrate. When the stretch velocity 0.1 nm/ps is imposed at the right end of the strip, an interface crack initiates and propagates very quickly. It seems that the above slider cannot sense the strain gradient field in time and stays at the original position in the whole process.

**Figure Captions**

Fig. S1 Typical snapshots for a relatively small nano-flake of 0.5nm long and 2.5nm wide on a graphene strip, which demonstrates a small nano-flake hardly sensing the gradient of the strain field. (a) Initially, the small nano-flake is put on the right end of the graphene strip; (b) After relaxation, the slider moves forward and stops at a position 1.5 nm far away from the right end of the graphene strip; (c) A constant velocity 0.01 nm/ps is added at the right end of the strip substrate and an interface crack initiates and propagates forward; (d) The slider keeps still even the crack tip is under it; (e) The crack tip propagates forward and surpasses the position that the slider stays, while the slider moves backwards together with the pulled-off segment of the graphene strip.

Fig. S2 Typical snapshots for a relatively large nano-flake of 20nm long and 2.5 nm wide on a graphene strip, which demonstrates a relatively slow moving velocity for a large nano-flake. (a) The large nano-flake lies at the right end of the graphene strip initially; (b) The slider will move a little to the left to find an equilibrium position; (c) A constant velocity 0.01 nm/ps is added at the right end of the strip substrate and an interface crack initiates and propagates forward; (d) The interface crack arrives near the left end of the slider and activates the slider motion; (e) The slider moves to the left as the crack tip propagates forward also; (f) The crack tip exceeds the slider; (g) The slider decelerates to find anther equilibrium position in the uniform strain field, which would not be activated again.

**
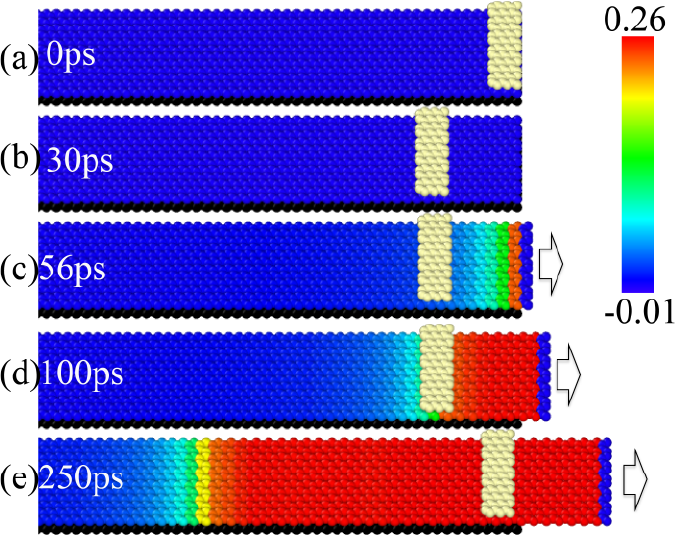
**

**Figure S1**

**
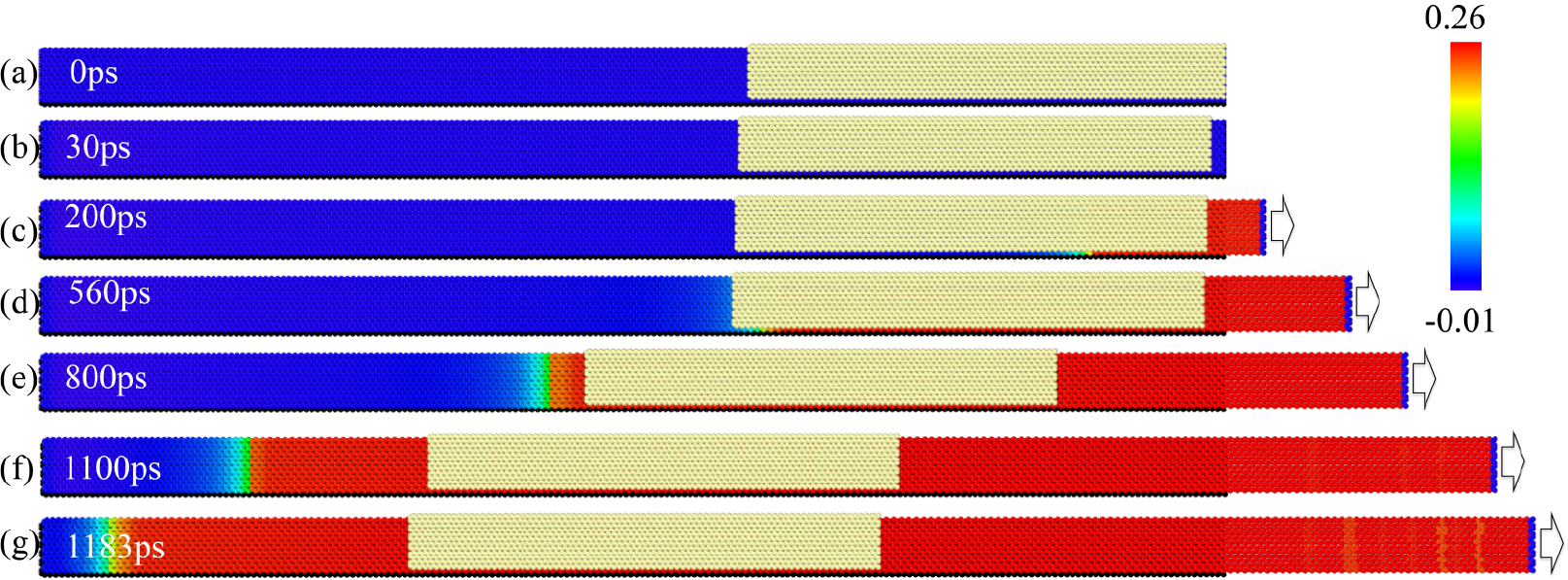
**

**Figure S2**
